# Review of dengue vectors in Cambodia: distribution, bionomics, vector competence, control and insecticide resistance

**DOI:** 10.1186/s13071-024-06481-5

**Published:** 2024-10-09

**Authors:** Bros Doeurk, Sébastien Marcombe, Pierre-Olivier Maquart, Sébastien Boyer

**Affiliations:** 1 Medical and Veterinary Entomology Unit, Institut Pasteur du Cambodge, PO Box 983, Phnom Penh, Cambodia; 2 Vector Control Consulting in Southeast Asia (VCC-SEA), Vientiane, Laos; 3 IRD, UMR 247 Evolution, Génome, Comportement, Ecologie,, Université Paris-Saclay, Gif-Sur-Yvette, France; 4 Ecology and Emergence of Arthropod-Borne Diseases, Institut Pasteur, Paris, France

**Keywords:** *Aedes aegypti*, *Aedes albopictus*, Behavior, Biology, Dengue, Bionomics, Ecology, Insecticide resistance, *Stegomyia*

## Abstract

**Background:**

Dengue fever is one of the most prevalent mosquito-borne diseases in Cambodia. Until now, no specific vaccine nor antiviral treatment exists the virus causing Dengue fever. Consequently, its prevention relies only on vector control strategies. However, efficient vector control in turn relies on a good knowledge of the biology of the vector species. Therefore, this study aims to provide the first review of the distribution, ecology, meteorological impacts, trophic behavior, vector competence, vector control and insecticide resistance of dengue vector species in Cambodia.

**Methods:**

A systematic search of the Google Scholar and PubMed databases was conducted for relevant published articles. Of the 610 published articles originally identified, 70 articles were ultimately selected for inclusion in this review. We also included new data from unpublished research conducted in Cambodia between 2017 and 2023 related to dengue vector bionomics.

**Results:**

Eleven *Aedes* (*Stegomyia*) mosquito species have been recorded in Cambodia, including a new species described in 2024. Four species are associated with dengue virus transmission, among which *Aedes aegypti* and *Ae. albopictus* are the main vectors and *Ae. malayensis* and *Ae. scutellaris* are considered to be potential vectors. *Aedes aegypti* and *Ae. albopictus* are present in all provinces of Cambodia. *Aedes albopictus* shows a preference for forest, rural and suburban areas, while *Ae. aegypti* is mostly found in urban and suburban areas. The distribution of these two species is also influenced by meteorological factors, seasonality and the availability of breeding habitats and blood meals. Both species are predominant during the rainy season, and their respective density is impacted by precipitation and temperature. *Aedes aegypti* is characterized as anthropophilic, while *Ae. albopictus* exhibits zooanthropophilic behavior, and both species have been observed to be predominantly diurnal. In addition, they were found to be highly resistant to the insecticides used in Cambodia for their control, such as temephos for larvae and deltamethrin and permethrin for adult mosquitoes.

**Conclusions:**

This review provides extensive and important knowledge on dengue vectors in Cambodia. This knowledge is derived not only from published research articles but also from many recent studies in Cambodia on the bionomics of dengue vector species. The review provides valuable information for use by public health authorities on dengue virus transmission and to develop better vector control strategies in the country.

**Graphical Abstract:**

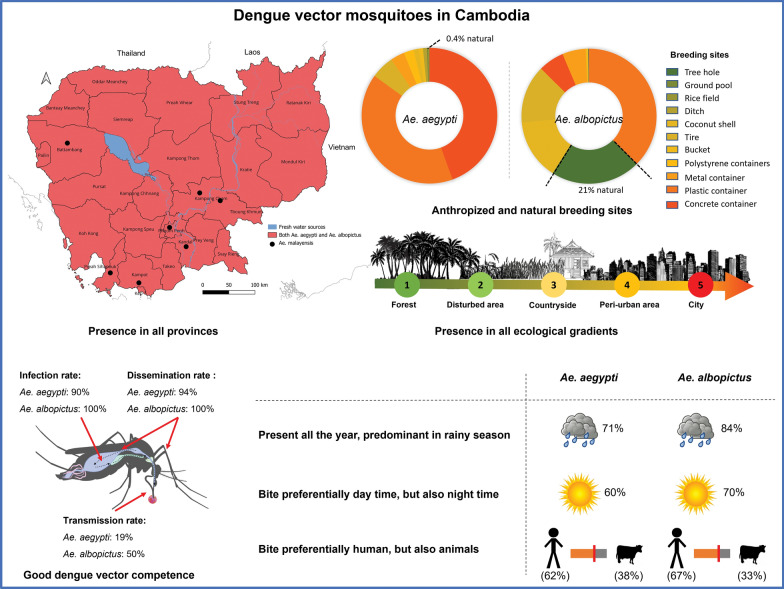

**Supplementary Information:**

The online version contains supplementary material available at 10.1186/s13071-024-06481-5.

## Background

Dengue virus is an arbovirus belonging to the genus* Flavivirus* in the* Flaviviridae* family. Currently, four serotypes (DENV-1, DENV-2, DENV-3, and DENV-4) are known [1, 2]. Although DENV-2 is known to have caused more deaths than the other serotypes, the first infection with DENV-1 or DENV-3 is considered to be more dangerous than infection with DENV-2 or DENV-4 [1, 2]. Dengue virus is the one of the most prevalent arboviruses, with 96 million cases of dengue fever (also referred to as dengue in this article) recorded yearly, representing a significant public health problem, leading to around 20,000 deaths annually worldwide [3, 4]. Approximately 3.9 billion people in 129 countries are currently at risk of contracting dengue, with 70% of global dengue cases reported in Asia [4–6]. Cases of dengue are most frequently recorded in children and young adults worldwide, compared with adults [3, 7], and the number of dengue cases have been documented to be dramatically increasing in urban and suburban areas in the last decades due to the increasing human population density and movement of people to and within cities [2].

Dengue viruses are transmitted through the bites of infected *Aedes* (*Stegomyia*) mosquitoes. Worldwide, two main vector species, *Aedes aegypti* (Linnaeus, 1762) and *Aedes albopictus* (Skuse, 1894)*,* are responsible for the transmission of dengue virus [8–10]. Geographically, these two species are widely distributed in many countries across different ecological gradients, including urban, suburban, rural and forested areas, where they breed in various natural and artificial containers [11, 12]. Specifically, *Ae. aegypti*, which originated in Africa [13], is widely distributed in tropical and subtropical regions of the world [14] while, in contrast, *Ae. albopictus,* known as the Asian tiger mosquito, originated from forested areas in Southeast Asia and is mainly distributed in tropical and temperate areas [15, 16]. In addition, *Aedes malayensis* (Colless, 1962) and *Aedes scutellaris* (Walker, 1858)*,* which are sylvatic mosquito species widely distributed in Southeast Asia, have been tested as competent vectors for the dengue virus in Thailand, Laos and Singapore [17] and are considered to be potential vectors of dengue in Cambodia [18, 19]. Due to their spatial distribution, *Ae. aegypti* and *Ae. albopictus* exhibit different genetic variations that impact the transmission of and infection by dengue virus [20–22]. Environmental factors, including climate variables, are critical factors involved in the spatial distribution of dengue vector mosquitoes that could contribute to the incidence of dengue transmission [11, 23, 24].

In Cambodia, the National Dengue Surveillance System was established in 1980, although the first dengue case was detected in the country in 1963 [25, 26]. Currently, dengue fever is regarded as one of the most critical mosquito-borne diseases in the country [25, 27]. All four dengue serotypes circulate each year, with the predominant serotype alternating between DENV-1, DENV-2 and DENV-3 over the past decades [25, 27, 28]. DENV-1 was identified as a minor dengue serotype co-circulating with other serotypes from 2000 until 2015, and DENV-3 was considered to be the main serotype causing a major outbreak of dengue in 2007 across Cambodian provinces [25, 28–31]. In Cambodia, dengue epidemics occur every 5 to 7 years, with three major epidemics occurring in 2007 (39,618 cases), 2012 (42,362 cases) and 2019 (68,597 cases) [26, 27, 30, 32, 33]. In 2019, most of the recorded dengue cases were in children under 15 years of age, with no observed association between incidence and sex (male/female) of patients [25, 27, 33]. Additionally, Cambodia has reported a high prevalence of asymptomatic patients, which is a factor in the global dynamics of dengue virus transmission in the country [27, 31].

Since there is no specific treatment or suitable vaccine available against the dengue virus, Cambodian public health authorities mainly rely on vector control strategies, with the implementation of physical, biological and/or chemical measures [4, 23, 34–41]. However, vector control depends on the availability of reliable information on the distribution, biology, ecology and behavior of dengue vector species, and such information is scarce and poorly characterized in Cambodia. The aim of this review was to collect exhaustive information on the distribution, ecology, meteorological impacts, trophic behavior, vector competence, vector control and insecticide resistance of dengue vector species in Cambodia (i.e. *Ae. aegypti*, *Ae. albopictus*, *Ae. malayensis* and *Ae. scutellaris*). To achieve this aim, we compiled data from published research articles and unpublished data produced by the Medical and Veterinary Entomology Unit at the Institut Pasteur du Cambodge (Phnom Penh, Cambodia).

## Methods

### Strategy

In this review, we characterize and discuss published and unpublished data separately to create a comprehensive database concerning dengue vector mosquitoes in Cambodia. The published database provides knowledge from previous research, supplemented by unpublished data currently being gathered in Cambodia on this topic.

### Published data

The PubMed and Google Scholar databases were systematically searched for relevant scientific articles using the keywords “*Aedes* dengue Cambodia,” “*Aedes* dengue Southeast Asia,” “*Aedes* dengue South East Asia,” “*Aedes* Cambodia,” “*Aedes* Southeast Asia,” “*Aedes* South East Asia,” “Dengue vector Cambodia,” “Dengue vector Southeast Asia,” “Dengue vector South East Asia,” “Dengue mosquito Cambodia,” “Dengue mosquito Southeast Asia,” “Dengue mosquito South East Asia,” “*Stegomyia* Cambodia,” “*Stegomyia* Southeast Asia,” “*Stegomyia* South East Asia,” “*Aedes albopictus* Cambodia,” “*Aedes albopictus* Southeast Asia,” “*Aedes albopictus* South East Asia,” “*Aedes aegypti* Cambodia,” “*Aedes aegypti* Southeast Asia,” “*Aedes aegypti* South East Asia,” “*Aedes malayensis* South East Asia,” “*Aedes scutellaris* Southeast Asia” and “*Aedes scutellaris* South East Asia”. We filtered the search using “allintitle” for Google Scholar and “Title/Abstract” for PubMed. We included studies investigating the distribution, ecology, meteorological impacts, trophic behavior, vector competence, vector control and insecticide resistance of dengue vector mosquitoes in Cambodia, and excluded studies on dengue virology and epidemiology, following the PRISMA guidelines for systematic reviews and meta-analyses [42].

### Unpublished data

The Medical and Veterinary Entomology Unit of the Institut Pasteur du Cambodge was established in 2018. In the years following its inception, the unit has initiated and participated in 16 international and national projects that have involved the sampling of mosquitoes in the country. Between 2017 and 2023, many entomological surveys were conducted across Cambodian provinces, covering different ecological types, including forests, mangroves, rice fields, agricultural areas and rural, peri-urban and urban areas, with > 600,000 mosquitoes collected so far in all 25 provinces. Various trapping methods were used for sample collection depending on the main objective of each project. For example, ovitraps were used for larvae collection at sampling sites to determine the relative density of *Aedes* mosquitoes, while line transects were used to characterize breeding habitats around the sampling sites. Adult mosquitoes were mostly collected using CDC light traps and BG-sentinel traps to monitor the relative density and diversity of mosquito species present in the sampling area [19, 43]. Studies using human landing catches (HLCs) [43] and double net traps (DNTs) were designed to investigate the behavior of adult mosquito species. DNTs were used for different baited traps, including human-, cow-, pig- and chicken-baited DNTs, with mosquitoes collected hourly from each site [44]. As reported in the Meteorological impacts section, we modeled the relative density of *Ae. aegypti* and *Ae. albopictus* with daily precipitation and temperature to gain an understanding of the meteorological impacts on mosquito density in Kampong Thom province. The climatic factors were classified by week (7 days) during a period varying from 1 to 4 weeks before the collection time. The Akaike information criterion stepwise procedure of the generalized linear model (GLM) using a Poisson distribution was used to select the best fit time of temperature and precipitation in association with the collection date. The final GLM model used negative binomial distribution, according to the over-dispersion of data.

In addition to field surveys, we conducted various experiments, such as developing methods for rearing mosquito larvae, testing for insecticide resistance, assessing vector competence and screening for pathogens [45, 46]. In the Vector competence section, we report a vector competence experiment conducted in 2023 involving *Ae. aegypti* and *Ae. albopictus* populations from Phnom Penh with DENV-1 circulating in Cambodia. The aim of this study was to gain an understanding of the infection (positive in the abdomen), dissemination (positive in the wings and head) and transmission (positive in the saliva) rates at 10 days post-infection (dpi) among the main vectors collected in urban areas and in the same location. In this experiment, 3- to 5-day-old female mosquitoes (F2 generation) were allowed to feed on artificial blood using a Hemotek system (Hemoteck Ltd., Blackburn, UK). After 10 dpi, mosquitoes were individually dissected, separating body parts to examine infection rates in the abdomen, dissemination rates in the wings and head and transmission rates in the saliva. During these experiments, mosquito species in Cambodia were identified using the illustration key from Thailand [47, 48]. These unpublished data were analyzed for this review to enhance existing knowledge and complement information currently available in published databases, providing a comprehensive snapshot of the current state of dengue vectors in Cambodia.

## Results

**Fig. 1:**
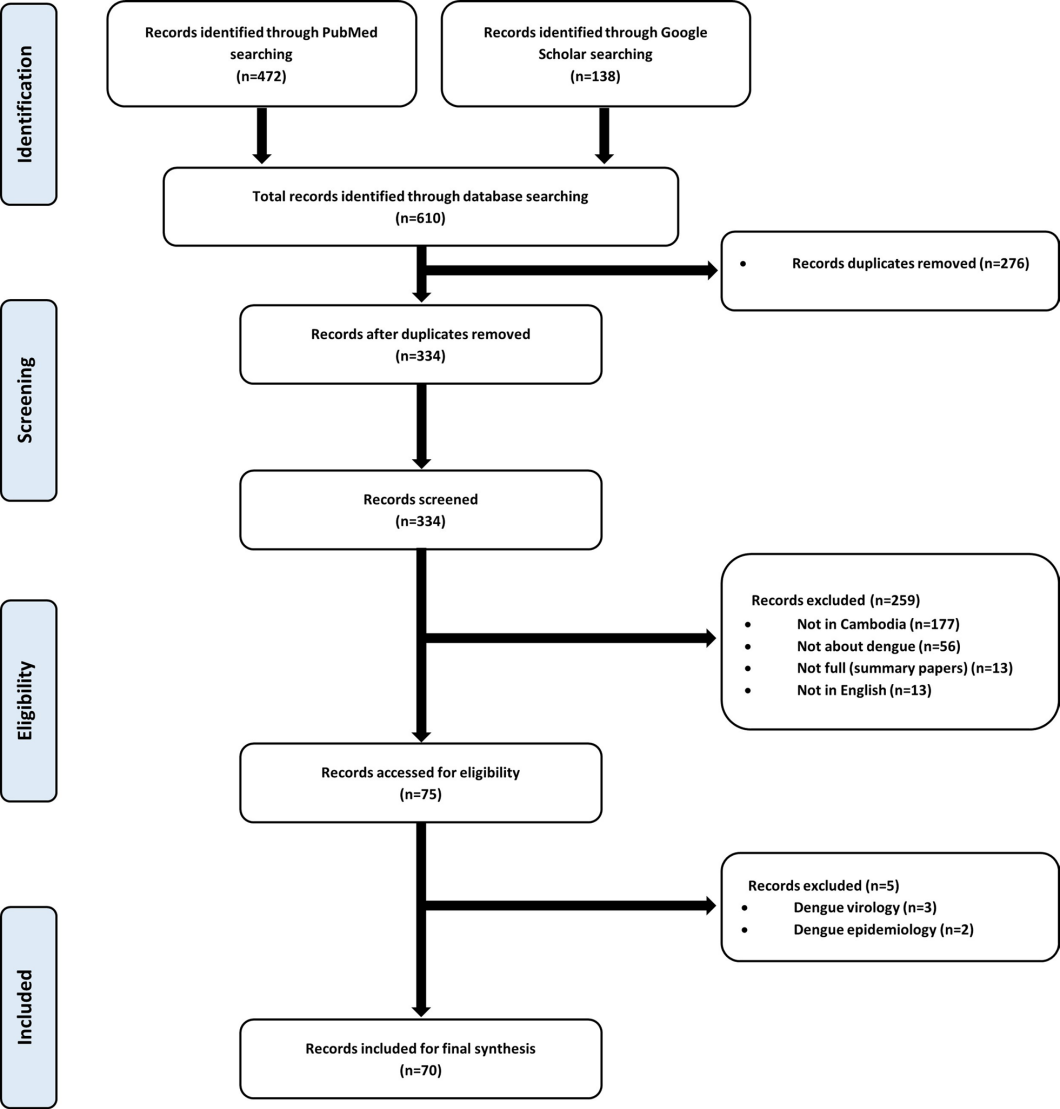
Flow chart of inclusion criteria for the systematic review and meta-analysis

In total, 610 articles were identified from the search of the Google Scholar and PubMed databases. Of these, 276 articles were duplicates and removed, leaving 334 items for screening. Of the 334 articles screened, 259 articles were excluded, leaving 75 articles eligible for inclusion in our review. The excluded articles included 177 non-Cambodian-related studies, 56 non-dengue vector studies, 13 articles not written in English and 13 conference proceedings. Among these 75 articles, five articles were further excluded: three focused only on virology studies and two were dengue epidemiological studies. Ultimately, 70 research articles were selected for inclusion in this review (Fig. 1).

Among the 11 *Aedes* (*Stegomyia*) species present in Cambodia, namely *Ae. aegypti, Ae. albopictus, Ae. annandalei, Ae. desmotes, Ae. gardnerii, Ae. malayensis, Ae. malikuli, Ae. pseudalbopictus, Ae. scutellaris, Ae. w-albus* and *Ae. unalom* [18, 43], we identified four species known to be involved in dengue virus transmission: *Ae. aegypti*, *Ae. albopictus*, *Ae. malayensis* and *Ae. scutellaris* [18, 19]. Of these four species, *Ae. aegypti* and *Ae. albopictus* were the most frequently studied and have been confirmed as vectors of dengue virus [15, 28, 31, 49]. However, the other two species, *Ae. malayensis* and *Ae. scutellaris*, are considered to be potential vectors of dengue virus serotypes [18, 19].

### Distribution

#### *Aedes* aegypti

##### Published data

**Fig. 2:**
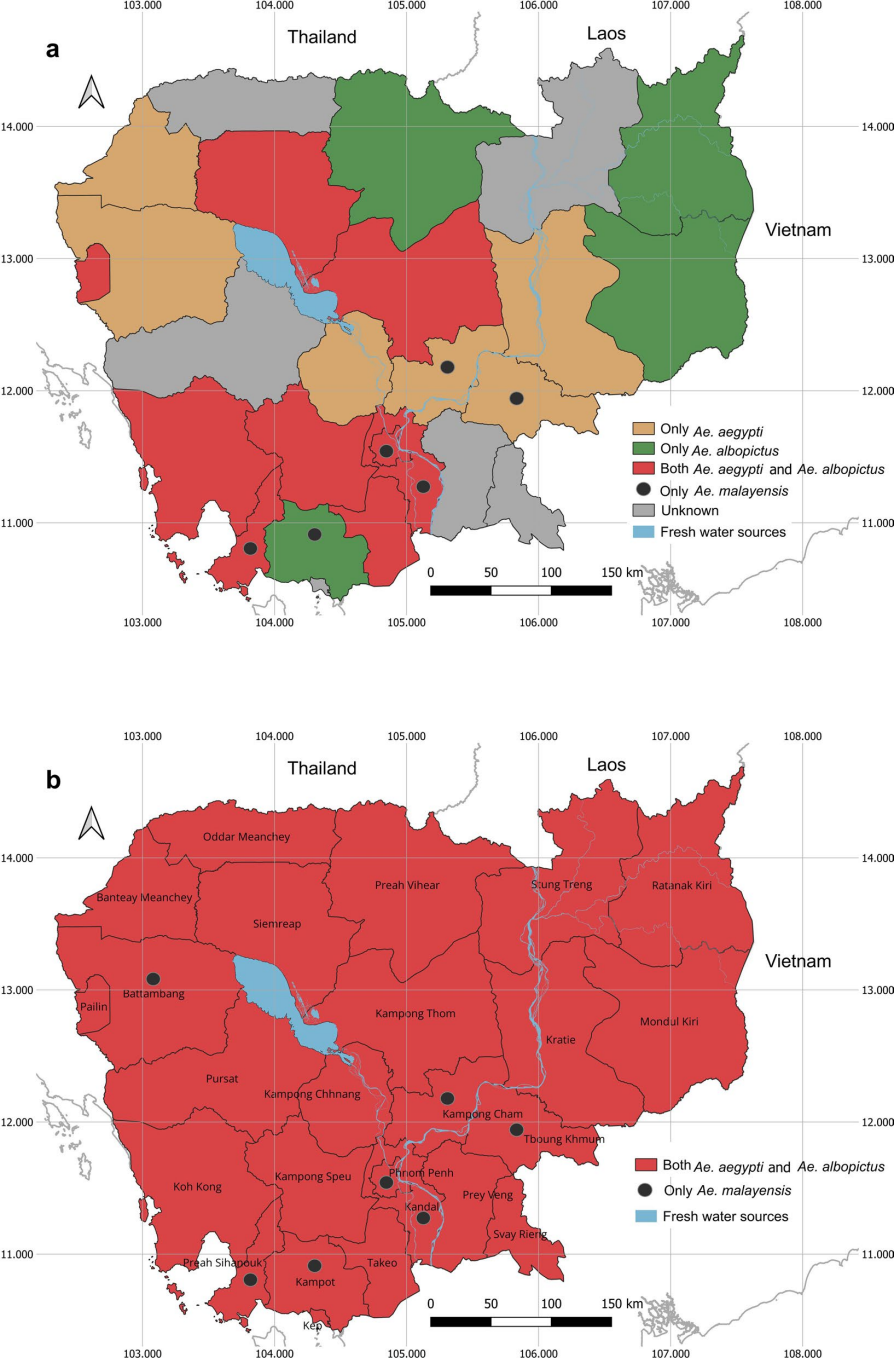
Map of Cambodia showing the distribution of dengue vectors across the 25 provinces. **a** Distribution of dengue vectors based on published data, **b** distribution of dengue vectors based on the current study

Across the 25 Cambodian provinces, *Ae. aegypti* was reported in 15 provinces between 1990 and 2023 (Fig. 2a): Banteay Meanchey, Battambang, Kampong Cham, Kampong Chhnang, Kampong Speu, Kampong Thom, Kandal, Koh Kong, Kratie, Pailin, Preah Sihanouk, Siem Reap, Takeo, Tboung Khmum and the capital city Phnom Penh [19, 44, 50–63]. Due to the widespread presence of *Ae. aegypti* in Cambodia, some studies were conducted to determine the genetic diversity of *Ae. aegypti* in the country [56, 63–66]. For example, *Ae. aegypti* mosquitoes showed significant genetic differentiation among the populations collected in Kampong Cham, Siem Reap, Preah Sihanouk and Phnom Penh [56]. In addition, genetic differentiation was detected even in mosquitoes collected within the same cities, except for Preah Sihanouk; the exception of Preah Sihanouk is probably due to it being the province with the heaviest rainfall in Cambodia, thereby creating more larval habitats and subsequently enhancing *Ae. aegypti* movement and genetic exchange in the area [56]. Genetic differentiation was associated with climatic factors, insecticide applications, urbanization and the season in which the samples were collected [56, 64, 67]. In Phnom Penh, *Ae. aegypti* mosquitoes collected from the city center had lower genetic differentiation than those from the suburbs [56, 64], indicating that people in suburban areas have the practice of storing rain water in jars, as well as an increased use of insecticide in such containers. Mosquitoes in these areas are likely to migrate to other locations in the search for breeding habitats as artificial sites disappear during the dry season [64]. In contrast, there is less genetic dispersal in the city center due to the constant availability of blood sources and artificial breeding habitats [64].

Among Southeast Asian countries, *Ae. aegypti* populations from Cambodia (Phnom Penh) and Vietnam (Ho Chi Minh City) show lower genetic differentiation, whereas populations from Thailand display higher genetic differences [20, 21]. The *Ae. aegypti* populations from Phnom Penh, Kratie and Battambang formed one genetic cluster, with the exception of one population located 50 km outside Battambang City, which showed genetic similarities to populations in Chiang Mai and Songkhla, Thailand [57, 58]. Genetic studies indicated that populations from West Africa (Guinea), East Africa (Uganda) and Asia (Cambodia, Singapore, Tahiti) are closely related based on mitochondrial haplotypes [68]. Another study showed that populations from Phnom Penh and Northeast Amazonia (Quixeramobim, Brazil) formed the same clade as those from Europa Island and Martinique (Riviere Salée) [69]. In addition, the Cambodian population of *Ae. aegypti* was found to be genetically similar to those in Venezuela, India, USA, Portugal and Cameroon [70].

##### Unpublished data

In addition to the results of this review, as well as those from our collections, *Ae. aegypti* was found in all provinces of Cambodia (Fig. 2b). Field results included the presence of *Ae. aegypti* recorded in 10 new provinces: Kampot, Kep, Mondulkiri, Oddar Meanchey, Preah Vihear, Prey Veng, Pursat, Ratanakiri, Stung Treng and Svay Rieng.

#### *Aedes albopictus*

##### Published data

*Aedes albopictus* is also widely distributed across Cambodia, with its presence reported in 13 provinces between 2001 and 2023 (Fig. 2a): Kampong Speu, Kampong Thom, Kampot, Kandal, Koh Kong, Mondulkiri, Pailin, Preah Sihanouk, Preah Vihear, Ratanakiri, Siem Reap, Takeo and the capital city Phnom Penh [44, 51–53, 55, 60–62, 71–73]. In Southeast Asia, *Ae. albopictus* populations in Cambodia, Thailand, Malaysia and Laos belong to the same genetic group [74, 75]. However, they are part of a genetic group that differs from populations in China, Japan and Korea, which are adapted to colder temperatures [74]. The genetic tree shows that *Ae. albopictus* populations in Southeast Asia, including Cambodia, are in the same genetic groups as those in Thailand (Chiang Mai) and Vietnam (Hanoi and Nha Trang), while populations in the USA (Jacksonville), Madagascar (Diego Suarez), France (MontSecret and Naintré), Hawaii (Oahu) and Réunion (La Possession and La Providence) are distinguishable [69, 76].

##### Unpublished data

In addition to the results reported in the reviewed articles, *Ae. albopictus* has been reported in all provinces of Cambodia based on our fieldwork, including 12 new provinces (Fig. 2b): Banteay Meanchey, Battambang, Kampong Cham, Kampong Chhnang, Kratie, Oddar Meanchey, Prey Veng, Pursat, Stung Treng and Svay Rieng.

#### Discussion

The two main dengue vector species, *Ae. aegypti* and *Ae. albopictus,* are distributed broadly throughout the world [14, 77]. In Cambodia, both species were previously not detected in some provinces [18], but they are currently present in all provinces of Cambodia (Fig. 2b). *Aedes aegypti* is well adapted to urban environments, while *Ae. albopictus* thrives in rural and forested areas. However, the presence of *Ae. albopictus* in urban areas indicates its adaptation to anthropogenic changes. Most *Ae. albopictus* populations from Cambodia are genetically similar to populations from other Southeast Asian countries [74, 75]. Understanding the genetics of *Ae. aegypti* mosquito populations is challenging due to their genetic similarities to populations in different continents [57, 58, 68, 70]. The genetic dispersal of these populations is likely due to the spread of the species via human migration and other human activities.

### Ecology: habitats, relative density and seasonality

#### *Aedes aegypti*

##### Published data

*Aedes aegypti* mosquitoes have been collected from various ecological gradients, including forested, rural, peri-urban and urban areas since 1990 in Cambodia [19, 50, 55, 60, 62], using a range of water containers, such as drums, water jars, concrete tanks, small pots, flower vases, tires, tins, broken pots/jars, dishes, trays, buckets, kettles and a number of unidentified containers [53, 59, 62, 67, 78]. Water storage jars were found to be the most prevalent breeding habitats for *Ae. aegypti* in Cambodia [67]. This mosquito species is widely distributed and occurs at high densities almost everywhere in the capital city of Phnom Penh [50, 55, 62]. In 2019, *Ae. aegypti* was collected from all collection sites across urban and suburban areas in Phnom Penh [55]. *Aedes aegypti* is predominant in the city but also found in rural villages, including farming communities [59, 62]. Interestingly, this species was also collected from forested areas in Kampong Speu province [60]. In one study, ovitraps were placed in 50 houses in Phnom Penh, with the results showing no significant difference in the number of eggs collected inside and outside the houses, respectively [54]. The results of many studies conducted in different provinces of Cambodia indicated that the highest density of this mosquito species occurs during the rainy season rather than the dry season [50, 53, 60, 71]; in terms of collection months, *Ae. aegypti* mosquitoes were most abundant in June and October [50, 71], corresponding to the rainy season in Cambodia.

##### Unpublished data

**Fig. 3:**
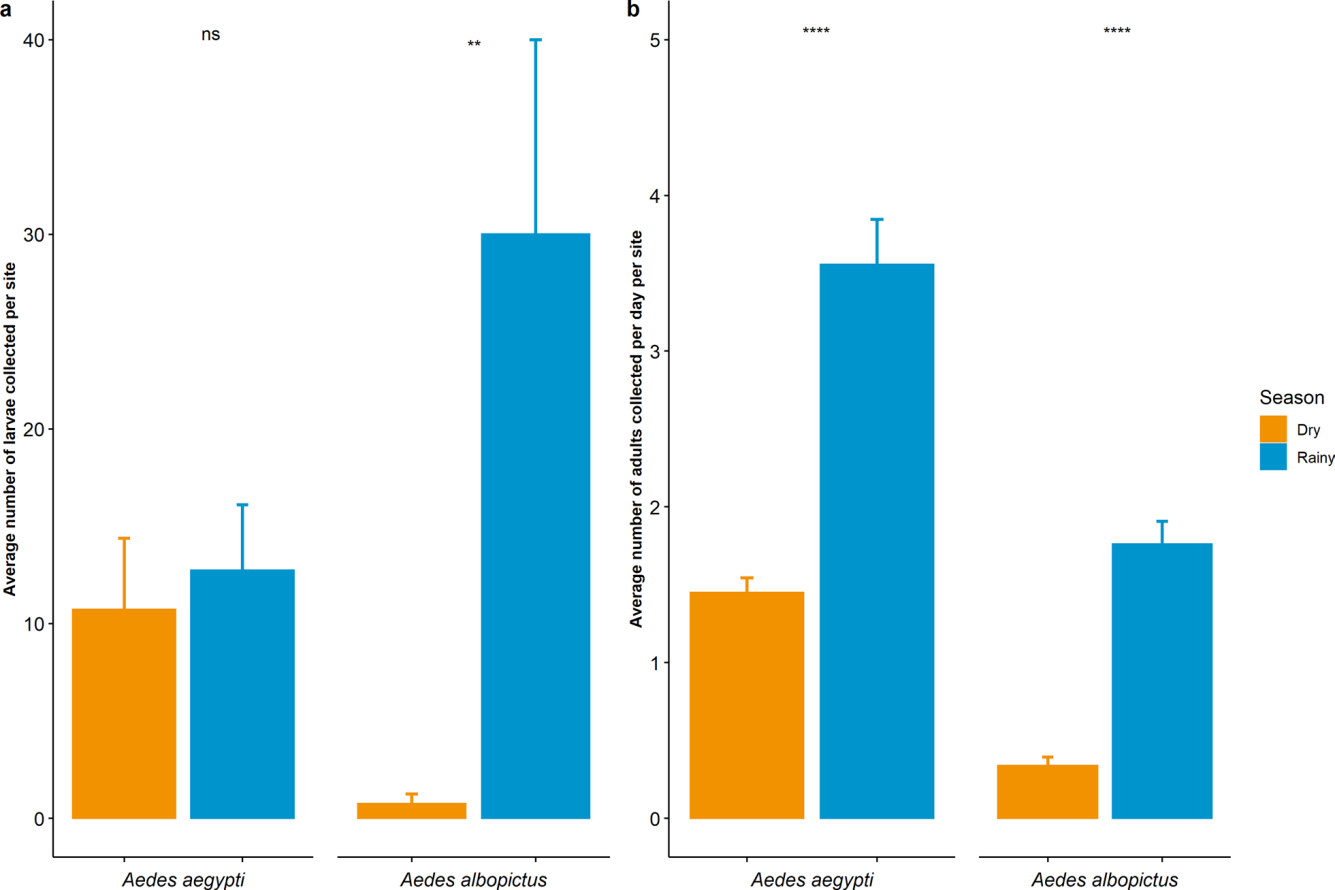
Average number of larvae and adults of *Aedes aegypti* and *Aedes albopictus* collected per site. **a** Average number and standard error of the number of mosquito larvae collected per site. **b** Average number and standard error of the number of adult mosquitoes collected per site per day. Asterisks above bars indicate significant differences between seasons by Student’s *t*-test at *P < 0.05, **P ≤ 0.01, ***P ≤ 0.001 and ****P ≤ 0.0001. ns, Not significantly different

Based on our data, *Ae. aegypti* mosquitoes are highly dominant in urban, peri-urban and rural areas. In particular, in the provinces of Kampong Thom, Pailin, Preah Sihanouk, Preah Vihear and Pursat, *Ae. aegypti* mosquitoes were collected from various ecological gradients, such as urban, peri-urban, rural, disturbed and forested areas. Among these five provinces, only in Kampong Thom province was this species present in forested, disturbed and rural areas, in sites close to human habitation, containing bamboo trees, rice fields and local villages. However, in all provinces, this species was collected in peri-urban and urban areas. During a 3-year study in Kampong Thom province from 2021 to 2023, mosquitoes were collected every 2 months from 40 sites across different habitats, including urban and residential areas, wetlands, wooded areas, river areas and rice fields. A total of 4597 *Ae. aegypti* mosquitoes were collected, of which 1314 individuals were collected in urban areas (28.6%), 1013 in wetland areas (22.0%), 658 in river areas (14.3%), 630 in wooded areas (13.7%), 568 in rural areas (12.4%) and 414 in rice field areas (9.5%) (Table 1). This study enhances our understanding of the seasonality of *Ae. aegypti* by showing that the relative density of mosquitoes collected during the rainy season was significantly higher than that during the dry season, with 71% (3265 individuals) and 29% (1332 individuals), respectively (*t*-test, *t*_(1107)_ = − 6.91, *P* < 0.0001; Fig. 3). In the same province and at the same collection sites, a 2-year survey (2022–2023) characterized the larval breeding habitats of dengue mosquitoes, which had not previously been studied in the country. In this study, mosquitoes were collected twice a year during the rainy and dry seasons, respectively. The authors identified a total of 978 *Ae. aegypti* larvae in the study, all found in various artificial containers, including 435 individuals in concrete containers (44.5%), 396 in plastic containers (40.5%), 54 in used tires (5.5%), 33 in metal containers (3.4%), 23 in polystyrene containers (2.4%), 12 in coconut shells (1.2%), 10 in buckets (1.0%), nine in ditches (0.9%) and two in rice fields (0.2%). A few individuals were also found in natural habitats, with three individuals found in tree holes (0.3%) and one in a ground pool (0.1%) (Table 2). In that study, 54% of *Ae. aegypti* larvae (537 individuals) were recorded during the rainy season and 46% (451 individuals) during the dry season. The tests displayed no statistically significant difference between the number of larvae collected per site during the rainy and dry seasons (*t*-test, *t*_(81)_ = − 0.40, *P* = 0.68; Fig. 3).

**Table 1:** The habitat landscapes of *Aedes aegypti* and *Aedes albopictus* mosquitoes collected in Kampong Thom province

Habitat landscapes	*Aedes aegypti* individuals,* n* (%)	*Aedes albopictus* individuals,* n *(%)
Urban	1314 (28.6)	8 (22.0)
Residential	568 (12.4)	93 (25.8)
River	658 (14.3)	18 (5.0)
Wooded	630 (13.7)	124 (34.3)
Wetland	1,013 (22.0)	75 (20.8)
Rice field	414 (9.0)	43 (11.9)

**Table 2:** Larval breeding habitats of *Aedes aegypti* and *Aedes albopictus*

Larval habitats	*Aedes aegypti* individuals,* n* (%)	*Aedes albopictus* individuals,* n* (%)
*Anthropized artificial*		
Concrete container	435 (44.5)	55 (6.2)
Plastic container	396 (40.5)	334 (37.5)
Polystyrene containers	23 (2.4)	1 (0.1)
Metal container	33 (3.4)	50 (5.6)
Bucket	10 (1.0)	4 (0.4)
Tire	54 (5.5)	124 (13.9)
*Anthropized natural*		
Coconut shell	12 (1.2)	129 (14.5)
Ditch	9 (0.9)	1 (0.1)
Rice field	2 (0.2)	0 (0.0)
*Natural*		
Tree hole	3 (0.3)	192 (21.5)
Ground pool	1 (0.1)	1 (0.1)

#### *Aedes albopictus*

##### Published data

*Aedes albopictus* has been recorded from various environments in Cambodia, including forested, rural, peri-urban and urban areas since 2007 [19, 52, 55, 60, 62, 73]. A study conducted on mosquitoes in forests across four provinces (Kampong Speu, Preah Vihear, Ratanakiri and Siem Reap) found *Ae. albopictus* to be the predominant species, recorded in collections from all provinces [60]. This study also showed that the relative abundance of this species was positively associated with low altitudes ranging from 75 to 401 m a.s.l. [60]. Similarly, *Ae. albopictus* was highly abundant in forests in Mondulkiri and Pailin provinces [52, 73]. *Aedes albopictus* mosquitoes were not only recorded in remote areas but were also recently collected in 38 of 40 urban and suburban locations in Phnom Penh in 2019 [55]. Across all breeding habitats, the immature stages of this species have been found in natural breeding habitats, such as tree holes, bamboo stumps and leaf axils, as well as in artificial containers [53, 62, 72]. Monthly dynamics graphs of *Ae. albopictus* showed an increase in number from March to October, corresponding to the end of the dry season up to the end of the rainy season [71].

##### Unpublished data

In addition to the published data from 2017, *Ae. albopictus* has been recorded in all types of environments, including the capital city of Phnom Penh in 2019. They have been found predominantly in natural habitats, such as forests, disturbed areas, hilly regions, bat caves, bamboo holes, grasslands, wooded areas, plantations, croplands and wetlands. However, this species was also found to be widely distributed in urban and peri-urban areas in the provinces of Kampong Thom, Pailin, Preah Sihanouk, Preah Vihear and Pursat. A total of 361 *Ae. albopictus* adult mosquitoes were collected during a 3-year study (2021–2023) carried out across different landscapes in Kampong Thom province, including 124 mosquitoes collected in wooded areas (34.3%), 93 in residential areas (25.8%), 75 in wetlands (20.8%), 43 in river areas (11.9%), 18 in rice fields (5.0%) and eight in urban areas (2.2%) (Table 1). The relative density of the collected adult mosquitoes was significantly higher during the rainy season (303 individuals, 84%) than during the dry season (58 individuals, 16%) (*t*-test, *t*_(221)_ = − 9.14*, P* < 0.0001; Fig. 3). In the same province and at the same collection sites, a 2-year survey from 2022 to 2023 characterized the larval breeding habitats of dengue mosquitoes. This study was conducted twice a year during the rainy and dry seasons. In terms of larval breeding habitats, 891 *Ae. albopictus* larvae were collected from various artificial containers, including 334 individuals in plastic containers (37.5%), 129 in coconut shells (14.5%), 124 in used tires (13.9%), 55 in concrete containers (6.2%), 50 in metal containers (5.6%), four in buckets (0.4%), one in a polystyrene container (0.1%) and one in a ditch (0.1%). Additionally, *Ae. albopictus* mosquitoes were found at high densities in natural larval habitats, with 129 individuals found in tree holes (20.4%) and one individual in ground pools (0.1%) (Table 2). Larvae were highly dominant during the rainy season, with 93% (916 individuals) collected during the rainy season and only 7% (23 individuals) collected during the dry season (*t*-test, *t*_(29)_ = − 2.92, *P* = 0.006; Fig. 3).

#### Discussion

Before 2019, *Ae. aegypti* mosquitoes were most commonly found in urban areas, while *Ae. albopictus* mosquitoes were predominant in forested areas [55]. However, the presence of *Ae. albopictus* mosquitoes has recently been recorded in many Cambodian cities, including the capital city of Phnom Penh, alongside *Ae. aegypti*. This finding is similar to those reported for Vietnam, where in 2011, *Ae. albopictus* was found to breed in both the urban and suburban areas [79, 80]. This increase in *Ae. albopictus* in cities is due to the adaptation of immature stages of *Ae. albopictus* to various breeding habitats, both artificial and natural. In contrast, *Ae. aegypti* mosquitoes are predominantly found in artificial habitats and only rarely found in natural breeding habitats. In terms of seasonality, *Ae. albopictus* is predominant during the rainy season, as increased precipitation supports an increase number of the natural breeding habitats preferred by this species. On the other hand, there is no seasonality for *Ae. aegypti*, as it mainly breeds in artificial containers that people use to store water throughout the year. Therefore, it is challenging to determine if these two mosquito species are present in the same place and whether there is competition between the two species in Cambodia.

### Meteorological impacts

#### *Aedes* aegypti

##### Published data

Meteorological variations are factors that impact the relative density and diversity of *Ae. aegypti* mosquitoes in Cambodia [60, 71, 81]. Rainfall is a key environmental component that both generates and expands breeding habitats for mosquito larval stages [56], while temperature influences the developmental rate of immature mosquitoes [60]. A mosquito survey was conducted in four Cambodian forests between 2020 and 2021 in Kampong Speu, Preah Vihear, Siem Reap and Ratanakiri provinces. In this study, the relative density of *Ae. aegypti* mosquitoes was modeled to study the impact of meteorological factors on the collected mosquitoes. The results showed that the average rainfall in the 4 weeks preceding the collection had a negative impact on the presence of *Ae. aegypti*, while the average temperature in the 2 weeks preceding the collection had a positive impact on the presence of the species [60]. By the end of the twenty-first century (2081-2100), the density of *Ae. aegypti* in Southeast Asian countries, including Cambodia, is predicted to rise due to increasing temperatures in the future, with the density expected to increase from 25% in areas where climate mitigation measures are implemented to 46% in areas without any such measures [71].

##### Unpublished data

We collected adult mosquitoes of* Ae. aegypti* over a 3-year period (2021–2023) and* Ae. aegypti* larvae over a 2-year period (2022–2023), at 40 sites spread across different landscapes in Kampong Thom province. The relative density of *Ae. aegypti* mosquitoes was modeled with daily precipitation and temperature to study the impact of meteorological factors on mosquito density in this province. The results showed that the relative density of larvae of this species was positively and statistically significantly impacted both by the temperature in the 7 days before the collection (GLM; estimate = 0.298, *Z* = 2.110, *P* = 0.034) and by the precipitation in the 7 days before the collection (GLM; estimate = 0.016, *Z* = 1.998, *P* = 0.045). Additionally, the density of adult *Ae. aegypti* mosquitoes was positively and statistically significantly impacted by the temperature in the 7 days before the sample collection (GLM; estimate = 0.093, *Z* = 4.056, *P* < 0.0001) and by the precipitation in the 21 days before the sample collection (GLM; estimate = 0.006, *Z* = 3.848, *P* < 0.0001) (Table 3).

**Table 3:** Summary of generalized linear models analyzing the impact of meteorological factors on the relative density of collected mosquitoes

Survey	Parameters	*Aedes aegypti*				*Aedes albopictus*			
		Time fit	Estimate	SE	*P*-value	Time fit	Estimate	SE	*P*-value
Larvae	Temperature	Week 1	0.298	0.141	0.034*	Week 1	− 0.699	0.236	0.003**
	Precipitation	Week 1	0.016	0.008	0.045*	Week 3	− 0.002	0.004	0.64
Adult	Temperature	Week 4	0.093	0.023	< 0.0001***	Week 3	0.074	0.044	0.092
	Precipitation	Week 1	0.006	0.001	< 0.0001***	Week 3	0.002	0.001	0.003**

*SE* Standard error

*, **, ***Significant effect by generalized linear models (GLM) at *P < 0.05, **P ≤ 0.01 and ***P ≤ 0.001

#### *Aedes albopictus*

##### Published data

The association between the population dynamics of *Ae. albopictus* and climate factors have been examined in Cambodia [71, 73, 81]. In 2013, a high density of this species was recorded in the forests of Mondulkiri province at an average temperature of 32.6 °C (minimum 32 °C, maximum 36 °C) and an average relative humidity of 75.4% (minimum 60%, maximum 87%) [73]. Although rainfall generally has a positive impact on the density of these mosquitoes, heavy rain during the day of collection significantly reduced the number of adult mosquitoes collected compared to days without heavy rain [73]. In another study carried out in the forests of Kampong Speu, Preah Vihear, Ratanakiri, and Siem Reap provinces between 2020 and 2021, the average temperature in the 4 weeks preceding the collection had a positive impact on the relative abundance of *Ae. albopictus* [60]. The same study also indicated that the abundance of this species is positively correlated with altitude due to the effect of temperature in land areas at a higher altitude [60]. A study conducted in Southeast Asian countries, including Cambodia, predicted that the relative density of *Ae. albopictus* will increase due to increasing temperatures in the future [71]. Consequently, the density of this species is expected to increase from 13% in areas with climate mitigation measures to 21% in areas without such measures by the end of the twenty-first century (2081–2100) [71].

##### Unpublished data

We collected adult mosquitoes of *Ae. albopictus* over a 3-year period (2021–2023) and *Ae. albopictus* larvae over a 2-year period (2022–2023), at 40 sites spread across different landscapes in Kampong Thom province. Daily temperature and precipitation factors were used to model the relative density of the collected mosquitoes. The results indicated that the relative density of mosquito larvae was negatively and statistically significantly associated with the temperature in the 7 days before the collection (GLM; estimate = − 0.699, Z = − 2.961, *P* = 0.003) while precipitation in 21 days before the collection was not significantly correlated to larval relative density (GLM; estimate = − 0.002, *Z* = − 0.467, *P* = 0.640). Additionally, the density of *Ae. albopictus* adult mosquitoes was positively and statistically significantly associated with the precipitation in the 21 days before the sample collection (GLM; estimate = 0.002, *Z* = 2.962, *P* = 0.003), while there was no significant association with the temperature in the 21 days before the collection date (GLM; estimate = 0.074, *Z* = 1.684, *P* = 0.092) (Table 3).

#### Discussion

The dynamics of dengue vector mosquitoes is highly dependent on climatic and meteorological conditions. Precipitation and temperature play a significant role in affecting the density of dengue vectors. Precipitation drives the availability of larval breeding habitats while temperature generally impacts the developmental rate of immature stages of mosquitoes [82, 83]. Most studies have investigated the influence of meteorological factors on the presence and abundance of *Ae. aegypti* and *Ae. albopictus* in Cambodia using prediction models [60, 71]. Nevertheless, there is an absence of scientific studies conducted in the country to define the impact of temperature on the development of dengue vector mosquitoes, and these need to be carried out in the future.

### Trophic behavior

#### *Aedes aegypti*

##### Published data

*Aedes aegypti* mosquitoes are known for their diurnal biting activity, and this behavior may contribute to the maintenance and transmission of the dengue virus in 24 schools in rural areas of Kampong Cham province [19]. A study conducted in 2012 and 2013 in the Mondulkiri and Pailin provinces of Cambodia using HLCs found that the biting behavior of *Ae. aegypti* mosquitoes was highest between 6:00 p.m. and 7:00 p.m. [52].

##### Unpublished data

In our mosquito biting behavior database, *Ae. aegypti* mosquitoes are generally considered to be anthropophilic as they preferentially feed on humans and only rarely attracted to domestic animals such as cows and chickens. In a study conducted in a small village (Roveak) in Mondulkiri province in 2020, sylvatic mosquitoes were collected outdoors at monthly and hourly intervals using human- and cow-baited DNTs. Analysis of the catches showed that > 60% of *Ae. aegypti* mosquitoes were collected with human baited traps, while < 40% were collected with cow baited traps. In another investigation conducted in a rural village in Kandal province, a few *Ae. aegypti* mosquitoes were collected from human and chicken traps. In 2021, monthly mosquito surveys were conducted in different habitat types using BG-sentinel traps placed inside and outside of houses with the aim to understand the biting behavior of this species (endo-exophagic behavior). A total of 2788 *Ae. aegypti* mosquitoes were collected from 40 houses in Kampong Thom province. The relative density of *Ae. aegypti* mosquitoes was significantly higher indoors (1612 individuals; 58%) than outdoors (1176; 42%) (t-test, *t*_(998)_ = 4.129, *P* < 0.0001). The combined results from several surveys investigating mosquito behavior using double nets revealed that *Ae. aegypti* mosquitoes were more active from 4:00 to 10:00 p.m., with the highest peak in biting activity at 7:00 p.m.

#### *Aedes albopictus*

##### Published data

*Aedes albopictus* was reported to exhibit diurnal activity [19], and the results of a study conducted in forested areas of Mondulkiri and Pailin provinces in 2012 and 2013 recorded a biting peak between 6:00 p.m. and 7:00 p.m. [52]. Another study conducted in a Mondulkiri forest in 2013 to investigate the biting behavior of forest mosquitoes found that the biting activity of *Ae. albopictus* increased significantly between 3:30 p.m. and 4:30 p.m. [73]. This study also highlighted that biting activity decreased when there was heavy rain at night or in the late evening on the day of collection [73]. Additionally, this species tended to preferentially land on the head of collectors compared to other body parts [73].

##### Unpublished data

In terms of behavior, *Ae. albopictus* mosquitoes in Cambodia exhibits a diverse and opportunistic feeding pattern, with a broad host choice, including humans and animals. During mosquito collection in a forest in Mondulkiri province in 2020, > 60% of *Ae. albopictus* mosquitoes were collected with human-baited traps, while < 40% were collected with cow-baited traps. Additionally, based on host preference studies in rural areas of Kandal province, this species is attracted to human-, cow- and pig-baited DNTs. In another field study carried out in Kampong Thom province, BG-sentinel traps were placed outside and inside of 40 houses across different ecological types. Based on monthly collections in 2021, *Ae. albopictus* was significantly more active outdoors (*n* = 73 individuals, 79%) than indoors (*n* = 19, 21%) (t-test, *t*_(98)_ = − 6.242, *P* < 0.0001). In Mondulkiri province, *Ae. albopictus* was observed to be more active in the forest during the daytime from 6:00 a.m. until 8:00 p.m., with the highest biting activity observed between 3:00 p.m. and 6:00 p.m.

#### Discussion

Our results show that *Ae. aegypti* mosquitoes exhibit anthropophilic behavior, feeding more indoors than outdoors. In contrast, *Ae. albopictus* mosquitoes are considered to be zooanthropophilic, as they prefer to feed on both humans and animals and prefer the outdoors. These results are consistent those of studies conducted elsewhere, particularly in a number of Asian countries [84–86]. In addition, the biting behavior of both dengue vector species is most active during the day, with peak activity in the evening between 3:00 p.m. and 6:00 p.m. This means that both species are able to transmit pathogens to humans anywhere and at any time. However, the variation in biting time between species may depend on the location (village or forest) and the availability of blood sources, which needs to be confirmed.

### Vector competence

#### *Aedes aegypti*

##### Published data

*Aedes aegypti* is a major vector species of dengue/dengue hemorrhagic fever [15, 18, 19, 28, 31, 62, 64, 87–89]. The vector competence of this mosquito species for dengue virus in Cambodia was identified in 2003 [64] and has been studied through oral infection with dengue virus strains circulating in Cambodia. In one vector competence study carried out in 2003, *Ae. aegypti* from a Phnom Penh population were susceptible to DENV-2 virus [64]. The infection rate depended on the genetic populations and seasonality of the collected colonies [64]. A lower infection rate was observed during the dry season (57.0–94.5%), while a higher infection rate was observed in strains collected during the rainy season (64.5–100%) [64]. The authors of another study conducted in 2019 found that the DENV-1 virus was competent in *Ae. aegypti* mosquitoes from the Phnom Penh population [28], with a very high infection rate of between 86.7% and 100% [28]. Through direct and indirect infection of *Ae. aegypti*, human participants successfully infected mosquitoes from 2 days before to 6 days after the onset of illness [31]. In addition, asymptomatic and pre-asymptomatic individuals transmitted dengue virus to* Ae. aegypti* mosquitoes at a significantly higher rate than symptomatic individuals [31].

##### Unpublished data

In 2023, a total of 31 *Ae. aegypti* mosquitoes from the Phnom Penh population were examined in a competence study with DENV-1 circulating in Cambodia. The results were analyzed at 10 dpi: six samples were positive for DENV-1 in the saliva (6 samples, 19%), 28 samples were positive in the wings and head (28 samples, 90%) and 29 samples were positive in the abdomen (29 samples, 94%) (Table 4). Based on these preliminary results, the infection rate of *Ae. aegypti* is very high, while the observed transmission rate is low for this population.

**Table 4:** Positivity results for *Aedes aegypti* and *Aedes albopictus* from Phnom Penh orally infected with dengue virus serotype 1 at 10 days post-infection

Mosquito species and sample	Number of samples	Positive for DENV-1 (*n*)	Negative for DENV-1 (*n*)	Positivity (%)
*Aedes aegypti*				
Abdomen	31	29	2	94
Wing and head	31	28	3	90
Saliva	31	6	25	19
*Aedes albopictus*				
Abdomen	12	12	0	100
Wing and head	12	12	0	100
Saliva	12	6	6	50

*DENV-1 *Dengue virus serotype 1

#### *Aedes albopictus*

##### Published data

*Aedes albopictus* is a commonly found species in Cambodia and has been identified as a vector species of the dengue virus in this country [18, 19]. The oral receptivity of dengue among *Ae. aegypti* and *Ae. albopictus* collected in Southeast Asia, including Cambodia (only *Ae. albopictus*), has been studied. In 2001, the first of such studies investigated the vector competence of *Ae. albopictus* in Cambodia with DENV-2 [49]. The results showed that *Ae. albopictus* was susceptible to DENV-2, but with very low infection rates (5.3–25.0%) in the Ta Promh strain collected from Angkor Wat temple at Siem Reap province [49, 50]. The study results did suggest, however, that the infection rate significantly increased with increasing generations of the species in the laboratory, with infection rates observed to increase up to 60.7% in the same strains of F5 generation mosquitoes [49].

##### Unpublished data

As a primary result, 12 *Ae. albopictus* individuals from the Phnom Penh population were examined in the vector competence study with DENV-1 circulating in Cambodia. The low number of samples was due to the high mortality and low blood feeding rate of mosquitoes during the experiment. Among the samples examined, six samples were positive for DENV-1 in the saliva (6 samples, 50%), 12 samples were positive in the wings and head (12 samples, 100%) and 12 samples were positive in the abdomen (12 samples, 100%) at 10 dpi (Table 4). We demonstrated that very high infection rates were obtained from *Ae. albopictus* from the Phnom Penh population strain.

#### Discussion

From the literature, both *Aedes* species are known as competent vectors of dengue in Cambodia [28, 31, 49, 50, 64]. *Aedes aegypti* is involved in urban transmission of dengue, while *Ae. albopictus* is involved in the rural transmission due to their distribution in urban and rural areas, respectively. Few studies have investigated the vector competence of the two species with dengue viruses, with the results indicating only the infection rates of each species from different locations in Cambodia [31, 49, 50, 64]. However, while *Ae. albopictus* is currently present in urban areas together with *Ae. aegypti* and they present in the same places, the different transmission rates of the two species are unknown. Based on our preliminary results of the vector competence experiment, the transmission rate of *Ae. albopictus* is higher than that of *Ae. aegypti.* This study was challenging as we had a low number of mosquitoes, and we examined the transmission only at 10 dpi. Therefore, it is very important that the topic of dengue vector competence be examined in future studies in Cambodia in order to consider the transmission effectiveness of the two species in the same location.

### Vector control

#### *Aedes* aegypti

##### Published data

In Cambodia, biophysical interventions (such as biological control, the covering of containers and solid waste management) and community engagement strategies (including education, training, communication and behavior change) have been significantly effective in reducing *Ae. aegypti* mosquitoes and dengue transmission [4, 34–41]. For example, the introduction of guppy fish (*Poecilia reticulata*) and encouraging the covering of containers have been demonstrated as powerful biological controls for *Ae. aegypti* larvae in domestic water storage containers in Cambodia [34–40]. Since the 1980s, carbamate, organophosphate, organochlorine and pyrethroid insecticides (such as deltamethrin and permethrin) have been used in the country to control adult mosquitoes, while temephos, *Bacillus thuringiensis israelensis* (*Bti*) and spinosad have been used to control *Ae. aegypti* larvae [40, 45, 46, 59, 90–93]. In addition, the National Dengue Control Program of Cambodia (NDCP) in the early 1990s distributed temephos larvicide under the trade name Abate to control the immature stages of *Ae. aegypti* [25, 94, 95]. Pyriproxyfen has also been effectively tested in Cambodia as a means to inhibit the adult emergence of *Ae. aegypti*, with an effectiveness rate of up to 90% [36–38, 96, 97]. In 2003, the pyriproxyfen formulation was tested under field conditions among water storage containers in Phnom Penh to inhibit adult *Ae. aegypti* emergence for 6 months during the main dengue transmission period. The results of this study suggested that pyriproxyfen was effective in inhibiting adult emergence at rates of between 87% and 95% in 2003 [96] and between 80.4% and 100% in 2005 [97]. In Cambodia, current vector control operations and policies conducted by NDCP involve the use of temephos against larvae and deltamethrin against adults [45, 46].

#### *Aedes albopictus*

##### Published data

Biological and insecticide control methods have been applied for *Aedes* mosquito control [37, 38, 40, 91], but to date no scientific publications have specifically addressed the control of *Ae. albopictus* mosquitoes in Cambodia.

#### Discussion

In Cambodia, many studies have tested the effectiveness of various insecticides (*Bti*, carbamate, pyriproxyfen) and community engagement strategies (destruction of breeding sites or the use of guppy fish) against *Ae. aegypti* [40, 59, 96]. Additionally, the effectiveness of *Wolbachia* strategies against *Ae. aegypti* in Southeast Asia [98] should be considered for developing an alternative vector control strategy in Cambodia. Therefore, public health authorities need to implement and monitor new vector control strategies against dengue vectors in Cambodia.

### Insecticide resistance

#### *Aedes* aegypti

##### Published data

The application of insecticides was initially thought to be effective in reducing dengue mosquitoes or cases, but insecticide resistance appears to be increasing in Cambodia [45, 46, 90, 91, 99, 100]. *Aedes aegypti* from Phnom Penh was recently found to show moderate resistance to temephos and spinosad [45, 91, 99, 100]. The results of other bioassay studies showed that *Ae. aegypti* larvae in the Phnom Penh, Battambang and Kampong Cham populations were resistant to temephos, with the exception being the populations in Siem Reap. The authors of two of these studies also reported that adult mosquitoes were highly resistant to permethrin while lower resistance was detected to deltamethrin [46, 99]. In addition, several knockdown resistance (*kdr*) mutations responsible for pyrethroid resistance have been detected in *Ae. aegypti* from Cambodia [101–103], with a high frequency (> 90%) of the L982W + F1534C and V1016G + F1534C substitutions in populations from Phnom Penh [101]. The prevalence of natural populations highly resistant to organophosphates was found in *Ae. aegypti* from Phnom Penh (29% mortality) and Kandal (26%) [103].

#### *Aedes albopictus*

##### Published data

A recent study in 2024 characterized the insecticide resistance of *Ae. albopictus* mosquitoes from Cambodia [104]. In this study, a total of 1468 adult female* Ae. albopictus* mosquitoes were tested, of which 728 individuals were collected from a rural area in Pailin province (12°49.848ʹN; 102°36.949ʹE) and 740 were collected from the capital city of Phnom Penh (11°30.717ʹN; 104°54.031ʹE). The adult bioassays were conducted using filter papers following WHO guidelines with specific concentrations of insecticides, such as deltamethrin at 0.03% and 0.015%, permethrin at 0.25%, malathion at 0.8% and DDT at 4%. Both *Ae. albopictus* populations showed high resistance to all tested insecticides (mortality < 90%), except for the Pailin population, which showed suspected resistance to DDT (92%). Specifically, the mortality rates of the populations from Pailin province and Phnom Penh City were, respectively, 17% and 35% with deltamethrin, 30% and 29% with deltamethrin, 78% and 79% with permethrin, 18% and 27% with malathion and 92% and 86% with DDT. Sequences of regions of the voltage gated sodium channel (*vgsc*) gene showed a lack of *kdr* mutations in the populations. These results suggest that resistance is likely due to metabolic resistance specifically involving cytochrome P450 monooxygenases in these two resistant populations [104].

#### Discussion

Insecticides have been effective in preventing dengue vectors in Cambodia. However, mosquitoes are showing resistance to the insecticides (larvicides and adulticides) used. Resistance to permethrin and deltamethrin has been observed in both *Ae. aegypti* and *Ae. albopictus* collected in different provinces in Cambodia [45, 46, 104]. While larvae of *Ae. aegypti* show resistance to temephos and spinosad, information on any insecticide resistance in larvae of *Ae. albopictus* is lacking in the country.

### Other potential vector species

In addition to the two main dengue vectors (*Ae. aegypti*, *Ae. albopictus*), *Ae. malayensis* and *Ae. scutellaris* are considered to be potential vectors of the dengue virus in Cambodia [18, 19]. *Aedes malayensis* mosquitoes have been found in Kampot, Sihanoukville and Kandal provinces, and in the capital city of Phnom Penh since 1972 [72], and were recorded in Kampong Cham and Tboung Khmum in 2018 [19] (Fig. 2a). In addition, this species was recorded in a new province, Battambang, based on our unpublished data in 2020 (Fig. 2b). The distribution of *Ae. scutellaris* in Cambodia is currently unknown [18]. Immature stages of *Ae. malayensis* have been found in spathes, bamboo stumps, coconut shells, artificial containers, tree holes, rock holes, rock pools and water containers [72]. *Aedes scutellaris* mosquitoes have also been collected from coconut shells and artificial containers [18]. In Southeast Asia, *Ae. malayensis* has been experimentally identified as a dengue vector in Thailand and Singapore and as a potential bridge vector species in Laos [17]. However, *Ae. malayensis* and *Ae. scutellaris* have not yet been confirmed as vectors of the dengue virus in Cambodia. Despite experimental evidence of vector competence in Southeast Asia indicating that *Ae. malayensis* and *Ae. scutellaris* are confirmed vectors of dengue, further research is needed to study the vector status of these species in Cambodia.

## Conclusions

This review highlights current knowledge of dengue vector mosquitoes in Cambodia, including their distribution, ecology, the meteorological impact, trophic behavior, vector competence, vector control and insecticide resistance. To date, four *Aedes* (*Stegomyia*) species have been identified as involved in dengue virus transmission in Cambodia, among which *Ae. aegypti* and *Ae. albopictus* are confirmed as the main vector species, as determined by competence studies conducted in the country. These two species have been more extensively studied in Cambodia compared to the potential vectors *Ae. malayensis* and *Ae. scutellaris*. Currently, the two primary dengue vectors, *Ae. aegypti* and *Ae. albopictus*, are widely distributed across all provinces of Cambodia. While the potential vector *Ae. malayensis* is known to be present in several provinces, *Ae. scutellaris* has been rarely recorded. The geographical distribution of these vectors is influenced by various factors, including meteorological conditions, seasonality, availability of larval breeding habitats and blood sources. *Aedes aegypti* and *Ae. albopictus* have adapted to anthrophogenic changes, as they can breed in various containers, both artificial and natural. Both species have diurnal activity patterns, with their blood feeding behavior occurring primarily during the daytime. In addition to *Ae. aegypti*, the high density and urban adaptation of *Ae. albopictus* mosquitoes, combined with their competence for dengue virus, likely contribute to the high prevalence of dengue cases in Cambodia. Despite the development of multiple vector control techniques against these vectors, some vector control methods have proven to be very difficult to implement effectively, especially in urban areas. Moreover, both species have shown significant resistance to commonly used insecticides in Cambodia, such as temephos, spinosad, deltamethrin and permethrin. Given that dengue is the most prevalent mosquito-borne disease in Cambodia, the efficacy and efficiency of these techniques need to be monitored by entomologists, public health authorities and all stakeholders. Understanding the bionomics of dengue vector species also provides a better understanding of the risk of dengue virus transmission in Cambodia. Our review, therefore, highlights the importance of developing effective prevention and control strategies to reduce the incidence of dengue fever in the region.

## Supplementary Information

**Additional file 1: Database.** Unpublished database analyzed during the review.

## Data Availability

All data published data reported in this review are available in the cited references. Unpublished data analyzed in this study are available in Additional file 1.
